# Characterization of the complete chloroplast genome of *Festuca sinensis* (Gramineae)

**DOI:** 10.1080/23802359.2019.1704192

**Published:** 2019-12-27

**Authors:** Chengxiang Ding, Quanmin Dong

**Affiliations:** a Academy of Animal and Veterinary, Qinghai University, Xining, China;; b Key Laboratory of Superior Forage Germplasm in the Qinghai-Tibetan Plateau, Xining, Qinghai Province, China

**Keywords:** Chloroplast genome, *Festuca sinensis*, genome assembly, phylogeny

## Abstract

The complete chloroplast genome from *Festuca sinensis*, an important perennial bunchgrass of the Gramineae, is determined in this study. The whole chloroplast genome sequence of *F. sinensis* has been characterized by Illumina pair-end sequencing. The circular genome is 134,177 bp long, containing a large single-copy region (LSC) of 79,367 bp and a small single-copy region (SSC) of 12,510 bp, which are separated by a pair of 21,150 bp inverted repeat regions (IRs). It encodes a total of 150 genes, including 93 protein-coding genes (75 PCG species), 49 tRNA genes (30 tRNA species) and eight ribosomal RNA genes (4 rRNA species). The most of gene species occur as a single copy, while 30 gene species occur in double copies. The overall A + T content of is 61.6%, while the corresponding values of the LSC, SSC, and IR regions are 63.7, 67.4, and 56.0%, respectively. Phylogenetic analysis suggested that *Festuca sinensis* was relatively close to *Festuca ovina* compared with other species of *Festuca* genera in Gramineae among the species analyzed. This complete chloroplast genome will provide valuable insight into conservation and exploitation efforts for this species as well as useful resources for studying the Gramineae phylogeny.

*Festuca sinensis* is an important perennial bunchgrass of the Gramineae mainly distributed in western and northern China. This plant has great potential to be widely utilized for forage grass in the alpine regions of China with a well adaptation such as drought, cold and salinity (Wang et al. 2006). The development and applications of *F. sinensis* plays an important role in the establishment of grassland (Zhang and Zhao 2002). In addition, the biology characters of *Neotyphodium* endophyte from *F. sinensis* have been well identified (Jin et al. 2009). It is indicated that the *Neotyphodium* endophyte may improve host grass competitive abilities by increasing seed germination success and enhancing host plant growth under water stress, salt and other environmental stress conditions (Siegel et al. 2012). Therefore, this species needs to be better studied and prioritized as an important exploitation target. Analyzing the chloroplast genome proved to be an efficient approach to shed light on plant molecular systematics (Moore et al. 2010; Zhang et al. 2011). In the present study, the complete chloroplast genome sequence of *Festuca sinensis* will contribute effective use and provide useful resources for better understanding the evolution of the whole grass family (Gramineae).

Genomic DNA was extracted from fresh leaves of an individual of *Festuca sinensis* collected from the Qinghai–Tibet plateau (Qinghai Province, China; the specimen was deposited at Qinghai University; accession number: DCX-2018-ZHYM1-8). The genomic library was sequenced on an Illumina Hiseq X Ten platform. 10 million high quality reads were assembled into complete chloroplast genome by using Velvet (Zerbino and Birney 2008). The assembled chloroplast genome was annotated using Geneious (Kearse et al. 2012). Then, submitted to GenBank (accession number: MK116525). The chloroplast sequence of *F. sinensis* was 134,177 bp, including the large single copy region (LSC, 79,367 bp), the small single-copy region (SSC, 12,510 bp), and a pair of 21,150 bp inverted repeat regions (IRs). The circular genome contained 150 genes, including 93 protein-coding genes (75 PCG species), eight ribosomal RNA genes (4 rRNA species) and 49 tRNA genes (30 tRNA species). The most of the gene species occurred in a single copy, while 30 gene species occurred in double copies, including four rRNA species (4.5S, 5S, 16S and 23S rRNA), 8 tRNA species and 18 PCG species. The overall A + T content of the circular genome was 61.6%, while the corresponding values of the LSC, SSC and IR regions were 63.7, 67.4 and 56.0%, respectively.

A neighbor-joining phylogenetic tree was constructed based on the concatenated coding sequences of chloroplast genomes for a panel of 11 species (see Figure 1 for details) with the program MEGA6 (Tamura et al. 2013). The phylogenetic analysis supported the traditional taxonomy of the family Gramineae at the genus level. The species *Festuca sinensis* was found to be relatively closely related to *Festuca ovina* compared with other species of *Festuca* genera in Gramineae (Figure 1). We believe that it will provide valuable insight into conservation and evolutionary histories for this important species.

**Figure 1.**
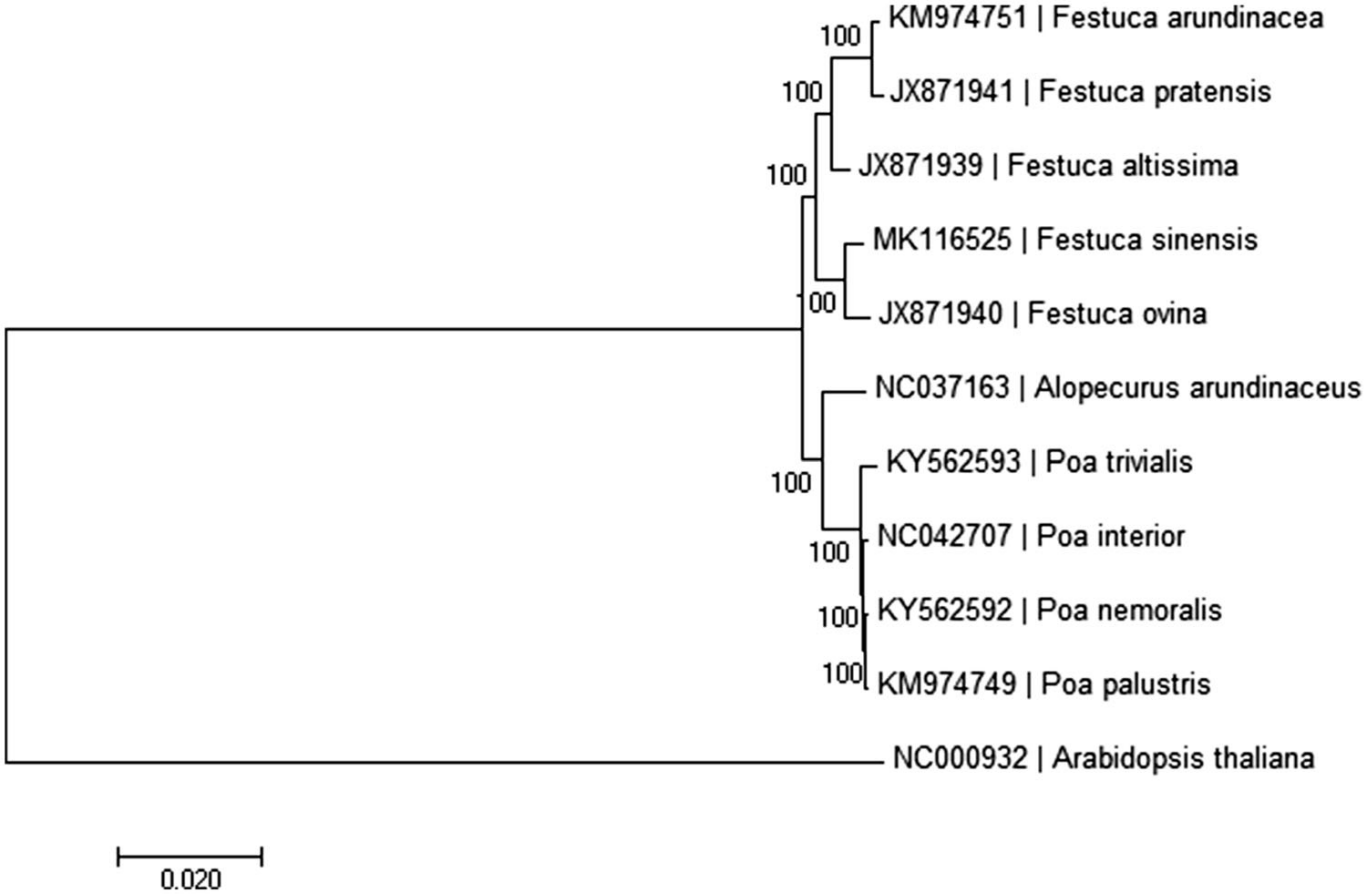
Neighbor-joining tree based on the complete chloroplast genome sequences of *Festuca sinensis* and related taxa within the Gramineae. The numbers on the branches are bootstrap values. The accession number of GenBank for each species is list in figure.
